# An Increase in Vascular Endothelial Growth Factor (VEGF) and VEGF Soluble Receptor-1 (sFlt-1) Are Associated with Early Recurrent Spontaneous Abortion

**DOI:** 10.1371/journal.pone.0075759

**Published:** 2013-09-30

**Authors:** Lihong Pang, Zhouling Wei, Ouyang Li, Rudian Huang, Junzhen Qin, Hongyan Chen, Xiaojing Fan, Zi-Jiang Chen

**Affiliations:** 1 Shandong University, Jinan, China; 2 Obstetrics, Gynecology and Reproductive Biology, The First Affiliated Hospital of GuangXi Medical University, Nanning, China; 3 Center for Reproductive Medicine, Provincial Hospital Affiliated to Shandong University, Jinan, China; 4 National Research Center for Assisted Reproductive Technology and Reproductive Genetics, Jinan, China; 5 The Key Laboratory for Reproductive Endocrinology of Ministry of Education, Jinan, China; 6 Shandong Provincial Key Laboratory of Reproductive Medicine, Jinan, China; Institute of Zoology, Chinese Academy of Sciences, China

## Abstract

Recurrent spontaneous abortion (RSA) is a health problem that affects approximately 1% to 5% reproductive age woman. Yet, in around half of these patients, the mechanism for RSA is unexplained. Recent studies have indicated that placental ischemia/hypoxia and endothelial dysfunction are important factors in miscarriage. Other studies have indicated that the level and expression of soluble FMS-like tyrosine kinase-1 (sFlt1) is increased under a hypoxic environment. However, decreased sFlt-1 in the maternal circulation during the first trimester has recently been proposed as a potential marker for identifying risk of pregnancy loss. In this prospective study clinical samples were obtained within a short time after the fetal death, protein expression and maternal serum levels of sFlt1 were assessed and compared to samples taken from those with normal pregnancies. Our results indicate that levels of VEGF and sFlt-1 are both increased in women during early pregnancy compared women that are not pregnant (p<0.05) indicating that VEGF and sFlt-1 are both associated with pregnancy. More importantly, we detected a significant (p<0.05) increase in sFlt1 and VEGF levels and expression in the RSA patients who suffered subsequent miscarriages compare to controls. These results demonstrate that there is likely a relationship between VEGF, sFlt-1 and RSA suggesting that the high levels and over expression of sFlt-1 and VEGF might be associated with the pathogenesis of RSA.

## Introduction

Recurrent spontaneous abortion (RSA) is defined as having had at least two consecutive embryo miscarriages within the first or early second trimester of pregnancy [1]. RSA affects approximately 1% to 5% of reproductive age women[2,3]. Various factors have been identified that are thought to play a role in about 50% of cases of RSA including genetic, endocrine, anatomical or autoimmune issues and infections. However the mechanism involved in the other 50% of RSAs remains unexplained (Unexplained RSA) [4,5]. Recent studies have indicated that placental ischemia/hypoxia and endothelial dysfunction may contribute to cases of RSA [6,7]. Yet the pathogenesis has not been fully elucidated and is still a matter of debate. Recent studies reported that sFlt-1 levels are markedly decreased in RSA patients [8,9]. However, in another study, expression levels of sFlt-1 mRNA and VEGF mRNA were found to be significantly higher in the chorionic villus tissue of women with RSA than in the control group [10].

Vascular endothelial growth factor (VEGF) is a multifunctional cytokine that is produced by a variety of cell types, including the placenta. VEGF modulates physiological and pathophysiological vascular development [11]. Several studies have indicated that the biological activity of VEGF is regulated by a soluble portion of the fms-like tyrosine kinase (Flt-1) receptor (sFlt-1), an endogenous inhibitor of VEGF, which may play an important role in endothelial dysfunction. Excess circulating sFlt-1 binds to VEGF with high affinity, thereby neutralizing it [12]. Clark et al. reported that sFlt-1 dramatically increases over the course of gestation and then dramatically falls soon after delivery. It has been hypothesized that the placenta is the primary source of this circulating anti-angiogenic factor[13].

Two-thirds of pregnancies that are lost to miscarriage are believed to be due to defective placentation associated with an absence of physiological changes in maternal spiral arteries [14]. Some recent studies suggest that a dysregulation of the angiogenic factors, VEGF and its solute receptor sFlt-1, may be involved in the pathophysiology of miscarriage [8,15]. Interestingly, other recent studies have shown that high levels of sFlt1 are associated with pre-eclampsia. In later pregnancy circulating sFlt-1 is increased in preeclampsia and also in other pregnancy complications hypoxia [16,17]. These results indicate that circulating sFlt-1 is elevated in preeclampsia during late gestation. The VEGF increase is much more dramatic, resulting in a high level of unbound or ‘‘free’’ VEGF in the system. A poorly vascularized (hypoxic) placenta stimulates excessive sFlt-1 production. The sFLt-1 is released into the maternal circulation throughout gestation, binding VEGF, which leads to endothelial dysfunction and hypertension. It has been well established that early embryo development in the human occurs in a hypoxic environment [11]. Tintu A et al. [18] reports that chronic hypoxia in early chick embryos resulted in increased sFlt-1 levels. However, the mechanisms by which VEGF and sFlt-1 induce miscarriage remain unclear. Specifically, the exact time when the over expression of VEGF and sFlt-1 induces miscarriage and/or the significance of high levels of VEGF and sFlt-1 cannot be determined. Thus we hypothesized that, in RSA patients, it is possible that a poorly vascularized (hypoxic) placenta stimulates excessive sFlt-1 and VEGF production throughout early gestation, stimulating additional sFlt-1 release, leading to endothelial dysfunction and miscarriage. A prospective study was conducted to test the hypothesis that under a hypoxic environment, sFlt-1 increases in the serum and chorionic villus during early pregnancy resulting in vascular-endothelial dysfunction and subsequent miscarriage.

## Materials and Methods

### Subjects and samples

A prospective case-controlled study was carried out between January 10, 2011 and December 10, 2012 at the Department of Obstetrics and Gynecology, The First Affiliated Hospital of Guangxi Medical University, Nanning, China. The institutional ethics committee of the First Affiliated Hospital of Guangxi Medical University approved the study protocol and written, informed consent was obtained from participants.

A total of 1140 women that had experienced the spontaneous abortion of two or more early pregnancies, prior to their current pregnancy, were enrolled in this study. All women were between 6 and 12 weeks pregnant at the time of spontaneous abortion. The study also included a control group of 50 women who induced abortions between weeks 6 and 12 of pregnancy but had no history of spontaneous abortion. Baseline characteristics of all women were recorded (Table 1). Pregnancy age for women in the miscarriage cohort was calculated from menstrual dates and for the controls, by either menstrual dates or dates of the first trimester ultrasound.

**Table 1:** Clinical Characteristics of subjects.

Characteristics	Control (N = 50)	RSA(N = 32)	P value
Age (years)	32.6 ± 2.1	33.2 ± 2.9	NS
Gestational Age (weeks) (ultrasound)	10.1 ± 1.91	10.9 ± 1.56	NS
No. of smoking (%)	0	0	NS
Pregnancy weeks (Menstruating)	10.3± 1.62	10.6 ± 1.82	NS
Median maternal weight kg (range)	51.5 (32.0-72.0)	52.1 (43.0-70.0)	NS
Median maternal BMI kg/m^2^ (range)	21.2 (15.6-33.1)	20.3 (16.6-27.3)	NS

For the miscarriage cohort the first prenatal clinic visit occurred between 6 and 12 weeks of gestation. Fetal cardiac activity was confirmed by ultrasound and women had no signs or symptoms of early pregnancy loss. All women enrolled were examined one time per week until the 12th week of gestation. Examinations included clinical symptoms, serum β-human chorionic gonadotropin (β-hCG) levels and ultrasound. A total of 32 patients, that subsequently miscarried, were included in this study. In all patients that miscarried, the clinic confirmed embryonic death and the ultrasound gestational week was matched to the pregnancy week.

All women enrolled were regularly menstruating, with a cycle length of < 32 days. Clinical details were recorded for each woman, and only patients who were certain of their menstrual dates were entered into the study group. Women with RSA, and those in the control group, had no history of adverse pregnancy outcomes, including preterm labour, had no current illnesses, did not use regular medication or smoke. Chromosome analyses were performed on both male and female partners in both the RSA patient and control groups; those with normal results were included in the study.

### Sample collection

Peripheral blood samples were taken from RSA patients and controls, prior to undergoing surgical uterine evacuation. Samples were centrifuged at 1200 rpm for 15 min after coagulation. The serum was then collected and stored at -80°C until analysis.

Chorionic villus tissues were collected after induced abortion in the control women and from RSA patients after subsequent spontaneous abortion. The specimen was washed in saline, then place in 10% buffered formalin and stored at room temperature for immunohistochemical (IHC) analysis. All samples were manually obtained, dissected, Formalin-fixed and paraffin-embedded.

### Immunoassay for sFlt-1 and VEGF

Commercial enzyme-linked immunosorbent assay (ELISA) kits (R&D Systems, Minneapolis, MN 55413, USA) were used, following the manufacturer’s instructions. The sensitivity of the assay was 5.01 pg/ml, with an intra-assay coefficient of variation of 2.6–3.8% and an inter-assay coefficient of variation of 7.0-8.1%.

### Immunohistochemistry

Each paraffin-embedded chorionic villus tissue sample was cut into 3 mm serial sections that were then immersed in distilled water 3 times, following routine methods. The sections were then mounted on microscope slides, air-dried and fixed in a mixture of 50% acetone and 50% methanol. The sections were then de-waxed with xylene, gradually hydrated with gradient alcohol and washed with PBS. Sections were incubated for 60 min with Mouse anti-human VEGF (ab2350;abcam,1:100, Cambridge, MA) / Rabbit anti-human soluble Flt1 (ab2350; abcam,1:250, Cambridge, MA). Following the PBS wash, sections were incubated for 30 min in a secondary biotinylated antibody (goat anti- rabbit Gig, ab128978, abcam, (Ready-To-Use), Cambridge, MA). Following washing, samples were coloured with 3,3-diaminobenzidin (DAB), kept at room temperature under a Microscope for 5 min. The positive color was brown or yellow. Following colouration, samples were washed with the distilled water and Hematoxylin stained. Sections were then dehydrated, cleared and mounted with neutral gums.

Samples from the negative control group analyzed using the same steps as described above, however the rat anti-human VEGF/ rabbit anti-human soluble Flt1 was replaced by PBS.

## Statistical Analysis

Statistical analysis was carried out using SPSS 14.0 software. Results are expressed as mean ± SD, or median (range), unless otherwise indicated. A p-value of < 0.05 was considered as statistically significant.

## Results

### Patient characteristics

The clinical characteristics of each group are presented in Table 1. There were no significant differences between the two groups.

### Serum VEGF and sFlt-1 levels

**Figure 1:**
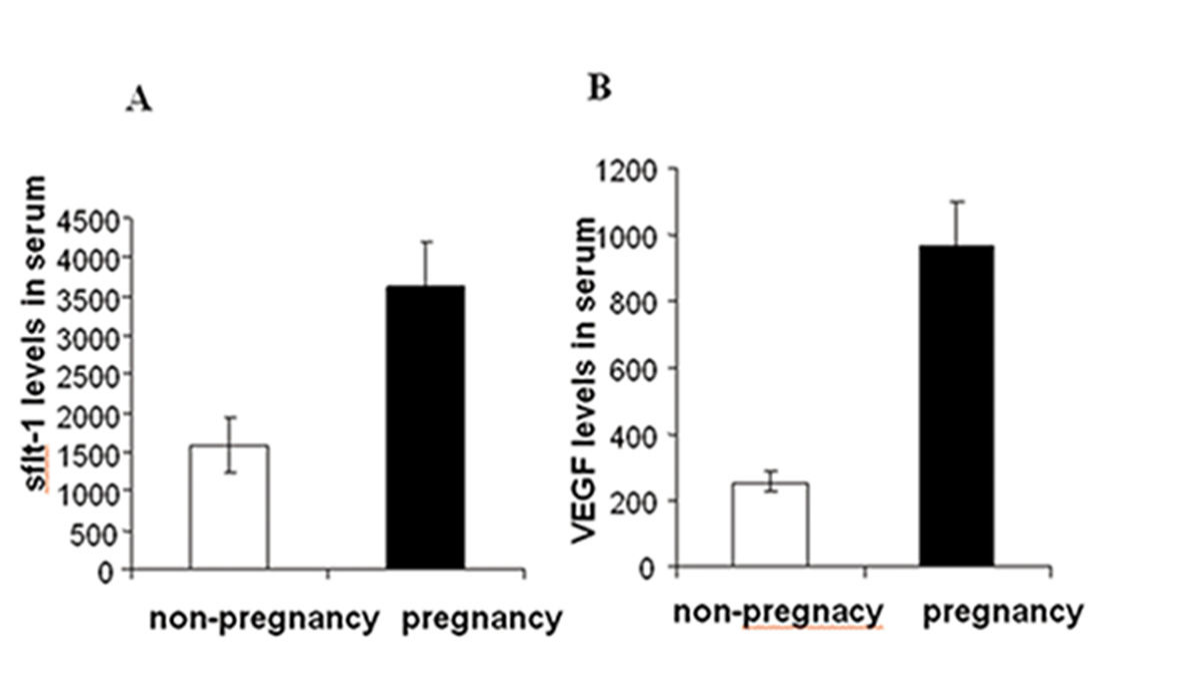
Levels of serum VEGF and sFlt-1 in pregnant and non-pregnant women. (**A**) The levels of sFlt-1 in women with normal pregnancies were significantly higher than in women that were not pregnant (*p*<0.05). (**B**) The levels of VEGF in women with normal pregnancies were significantly higher than in women that were not pregnant (*p*<0.001).

**Figure 2:**
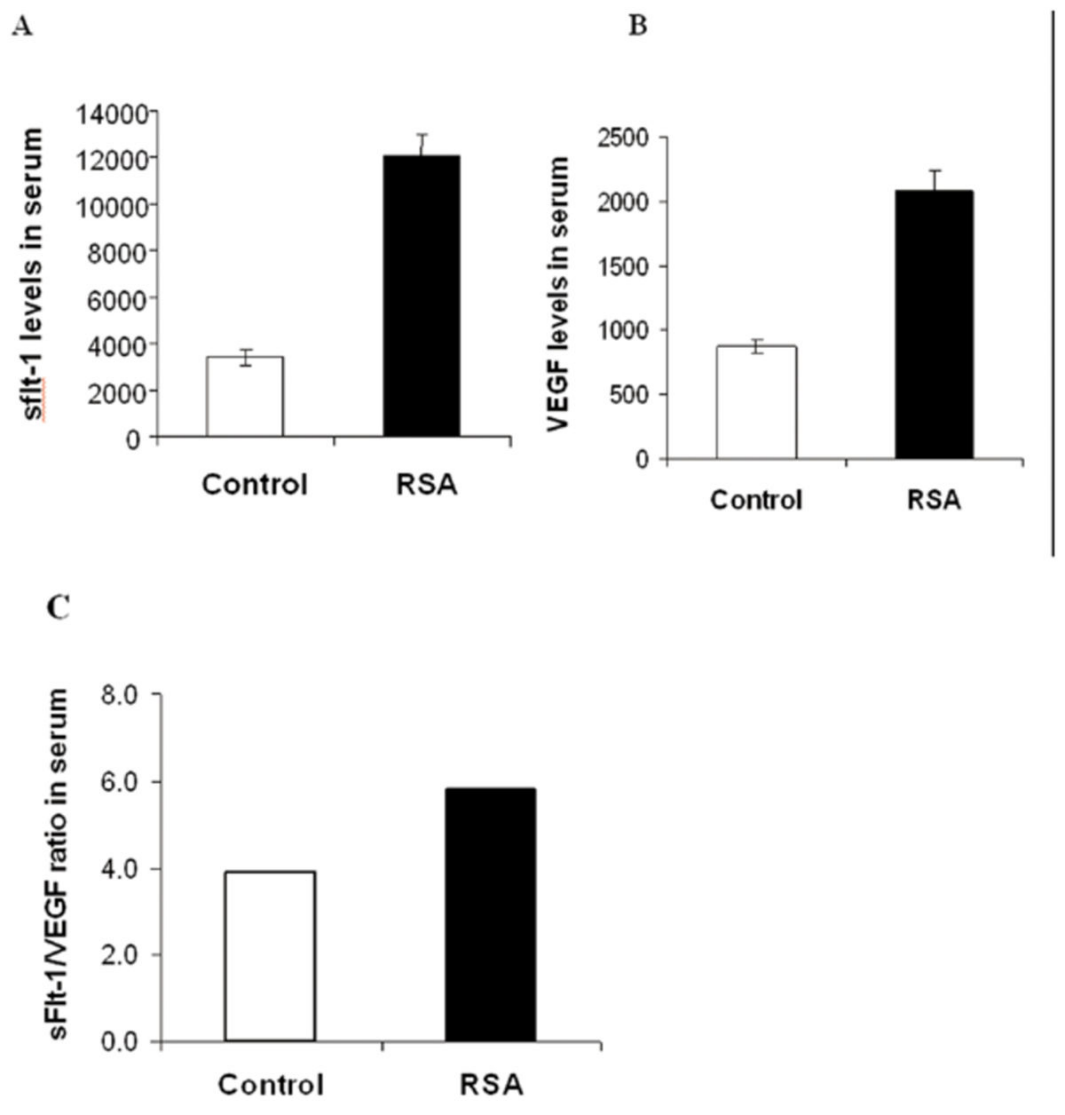
Levels of serum VEGF and sFlt-1 in RSA patients and normal pregnancy women. (**A**) The levels of sFlt-1 in the RSA group were significantly higher than in the control group (*p*<0.0001). (**B**) The levels of VEGF in the RSA group were significantly higher than in the control group (*p*<0.0001). (**C**) The ratio of sFlt-1/VEGF in serum.

The mean sFlt-1 level was higher in normal pregnancy group compared to the non-pregnant group (3631.742±3000.0891 pg/ml vs. 1587.001±1678.2486 pg/ml,P<0.05, Figure 1. A). The mean VEGF level was significantly higher in the normal pregnancy compared to the non-pregnant group (969.6429±696.9974 pg/ml vs. 253.8277±137.0413 pg/ml,P<0.001, Figure 1. B). The mean sFlt1 concentration was significantly higher in women with RSA than in women with early normal pregnancy (12047±946 pg/ml vs. 3407±354 pg/ml, P<0.0001, Figure 2. A). The VEGF concentration was also significantly higher in RSA group compared to the normal pregnancy group (2072±166 pg/ml vs. 874±56 pg/ml, P<0.0001, Figure 2. B). The sFlt-1/sFlt-1/VEGF ratio in serum was significantly increased in RSA women compared with normal pregnancy women (Figure 2. C).

### Immunohistochemistry studies

The immunohistochemical expression of biomarkers was evaluated using an OlympusBH2 microscope (10× ocular and 40× objective lenses) and an ocular lattice (area 0.092 mm^2^) with 100 points composed of 10 horizontal and 10 vertical test lines superimposed on the test field to be measured. A total area of 1.84 mm^2^ was evaluated for each sample. Immunohistochemical analyses of all antigens investigated were performed by determining the percentage of positively stained cells (trophoblastic and interstitial cells) in all fields counted (10 fields for each specimen). Immunohistochemical expression data are expressed as mean ± standard error (mean ± sem) values.

**Figure 3:**
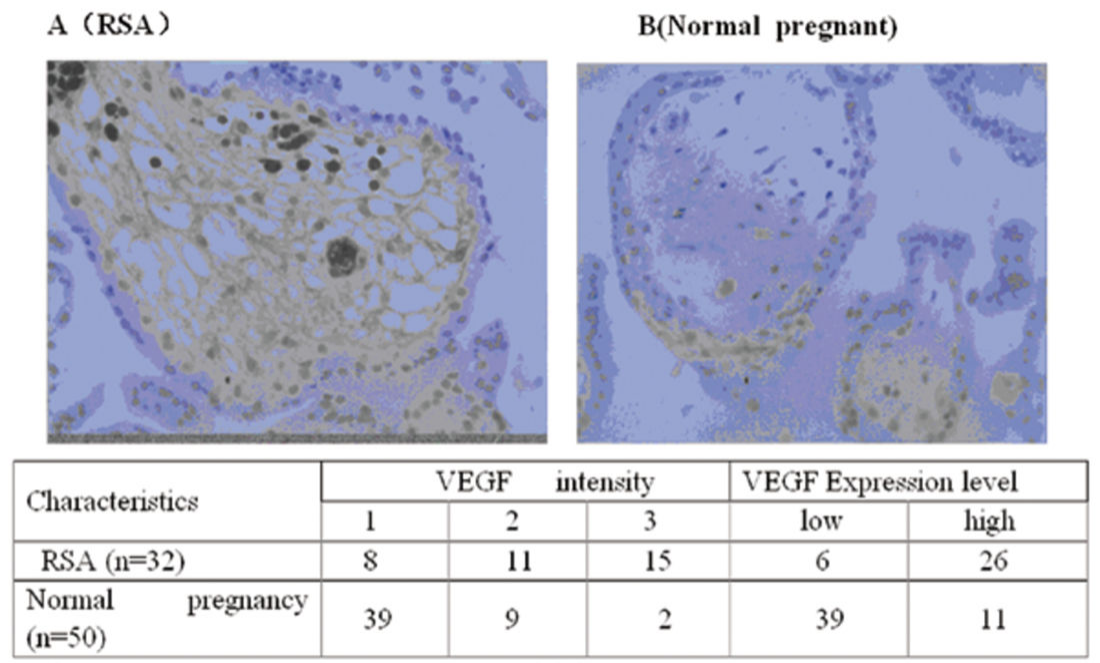
Immunohistochemistry for VEGF protein expression in chorionic villus. VEGF protein was expressed in the chorionic villus located in the cytoplasm (Original magnification ×600. VEGF intensity by immunohistochemistry staining was scored as following: 1=weak staining; 2=mild staining and 3=dark staining. *Based on the score of VEGF intensity, cases were classified into VEGF low-expression (low) for those cases in which of VEGF intensity scores were ≤1 and VEGF high-expression (high) for those cases in which of VEGF intensity scores were ≥2. Scores were generated and analyzed by LTQ-Orbitrap mass spectrometry. We found that the chorionic villus in RSA women that subsequently miscarried showed high expression of VEGF (n =24) (A), whereas this tissue showed low expression of VEGF during early pregnancy in women with normal pregnancies (n =16) (B).

**Figure 4:**
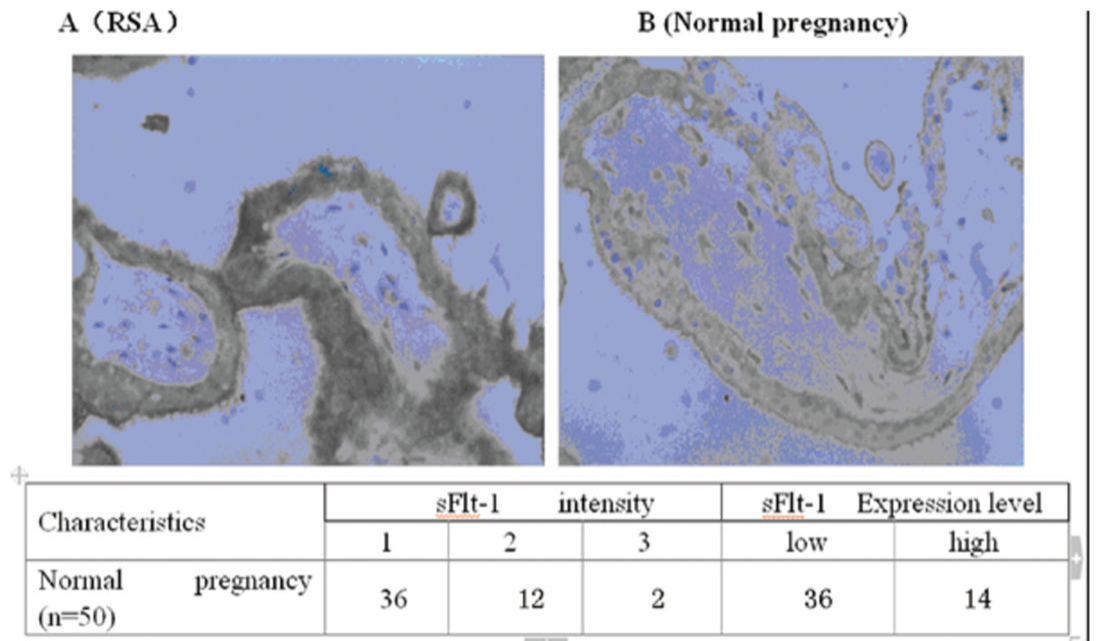
Immunohistochemistry for sFlt-1 protein expression in chorionic villus. sFlt-1 protein was expressed in the chorionic villus located in the cytoplasm (Original magnification ×600. sFlt-1 intensity by immunohistochemistry staining was scored as following: 1=weak staining; 2=mild staining and 3=dark staining. *Based on the score of sFlt-1 intensity, cases were classified into sFlt-1 low-expression (low) for those cases in which of sFlt-1 intensity scores were ≤1 and sFlt-1 high-expression (high) for those cases in which of sFlt-1 intensity scores were ≥2. Scores were generated and analyzed by LTQ-Orbitrap mass spectrometry. We found that the chorionic villus of RSA women that subsequently miscarried showed a high expression of sFlt-1 (n =26) (A), whereas this tissue showed a low expression of sFlt-1 during early pregnancy in women with normal pregnancies (n =18) (B).

The pattern expression of VEGF and sFlt-1 proteins by immunohistochemistry is shown in Figures 3 and 4 [19]. VEGF and sFlt-1 presented cytoplasmic localization. The samples analyzed were from RSA patients (n =32) and normal early pregnancy women (n =50). We found that women in the RSA group that subsequent miscarried had high VEGF and sFlt-1 protein expression in the cytoplasm (81.25% and 84.38%, respectively), while cytoplasmic expression of VEGF and sFlt-1 proteins were 22% and 28%, respectively in women with normal pregnancies. The observed difference in the VEGF and sFlt-1 protein expression between these two groups was statistically significant (*P* < 0.05).

Taken together, results of immunohistochemistry for VEGF and sFlt-1 expression in chorionic villus tissues showed that in RSA patients had a high expression compared to the normal pregnancy controls (p<0.05) (Figures 3 and 4). These results suggest that high expression is correlated with the risk of RSA.

## Discussion

In this prospective study we found a high level of expression of VEGF and sFlt-1 in various tissues and in the serum of RSA patients that subsequently miscarried, compared to controls. This indicates that there is a relationship between early RSA and VEGF and sFlt-1 and suggests that over-expression and high levels of the sFlt-1 and VEGF may be associated with the pathogenesis of RSA.

It has been reported that early stages of placental development occur under relatively hypoxic conditions and that specific placental proteins are regulated by intrauterine O_2_ tension [7]. We found high levels and expression of sFlt-1 and VEGF in the pregnant women compared to non-pregnant women, indicating that sFlt-1 expression in trophoblasts is a response to chronic hypoxia. Thus, it is suggested that, to a certain degree, the placenta favors invasive cytotrophoblast proliferation under conditions of chronic hypoxia [20]. VEGF and its receptor, sFlt-1, play a fundamental role in cytotrophoblast differentiation and survival during placentation and invading cytotrophoblasts are pivotal in the transformation of the maternal uterine circulation. In a recent study it was reported that increased sFlt-1 secretion was associated with reduced cytotrophoblast invasion in vitro [21]. Some studies have shown that placental hypoxia is regarded as a key factor in the pathogenesis of preeclampsia. It has been reported that uterus-placental ischemia can result in an elevated sFlt-1 level, which indicates a close relationship between the placental ischemia/hypoxia and over-expression of sFlt-1 [17,22].

Although high levels of sFlt1 are associated with pre-eclampsia in later pregnancy [20], in normal first trimester pregnancies, high levels of sFlt1 are believed to be physiological and a result of excessive placental production under hypoxic conditions [20]. Yet few studies have examined the relationship between sFlt1 and RSA, thus the connection between the two is not clear and is subject to debate. Two studies [12,13] have recently reported that there is a several-fold increase in levels of sFlt1 in early pregnancy and that these levels are markedly decreased in those patients at high risk for miscarriage who subsequently have a miscarriage, and that sFlt1 may be a potential biomarkers for indication of early pregnancy loss. However, a study by Kaitu’u-Lino TJ et al. indicates that sFlt1 is unlikely to be a useful predictor of miscarriage in the first trimester clinically, since sFlt-1 has a poor sensitivity and specificity for predicting miscarriage in asymptomatic women [14]. Results in the present study differ from those previously reported, as other researchers have shown that VEGF and sFlt-1 decrease after miscarriage. Conclusions regarding causes of miscarriage that are based on histopathological studies of abortion tissue are often questioned since fetal death may occur days before evacuation. During this ‘retention time’, vascular (tissue) may be injured and disappear due to apoptotic reactions. Until now, no effect has been reported regarding the effect of retention time on the outcome of vascular parameters. The current study shows that, in RSA patients that have subsequent miscarriages, both VEGF and sFlt-1 levels and expression increase compared to the controls in the short-term, after fetal loss. Although circulating VEGF and sFlt-1 is elevated in RSA patients, the sFlt-1 increase is much more dramatic resulting in a high level of unbound VEGF in the system (sFlt-1/VEGF ratio in serum). Our results indicate that it is possible that a poorly vascularized (hypoxic) placenta stimulates excessive sFlt-1 and VEGF production and that these are released into the maternal circulation shortly after miscarriage. The high sFlt-1 level, in particular, leads to endothelial dysfunction and RSA. It may be that both VEGF and sFlt-1 are involved in the endothelial dysfunction in RSA. 

In conclusion, this report presents evidence indicating that, in RSA patients, there is a correlation of overdressing of VEGF and sFlt-1 in the chorionic villus tissue. These observations suggest that VEGF and its soluble receptor, sFlt-1, play important roles in the pathogenesis of RSA.
